# Infrared dielectric metamaterials from high refractive index chalcogenides

**DOI:** 10.1038/s41467-020-15444-0

**Published:** 2020-04-03

**Authors:** H. N. S. Krishnamoorthy, G. Adamo, J. Yin, V. Savinov, N. I. Zheludev, C. Soci

**Affiliations:** 1 Centre for Disruptive Photonic Technologies, TPI, SPMS, Nanyang Technological University, Singapore, 637371 Singapore; 2 Optoelectronics Research Centre & Centre for Photonic Metamaterials, University of Southampton, London, SO17 1BJ UK

**Keywords:** Nanophotonics and plasmonics, Mid-infrared photonics, Metamaterials

## Abstract

High-index dielectric materials are in great demand for nanophotonic devices and applications, from ultrathin optical elements to metal-free sub-diffraction light confinement and waveguiding. Here we show that chalcogenide topological insulators are particularly apt candidates for dielectric nanophotonics architectures in the infrared spectral range, by reporting metamaterial resonances in chalcogenide crystals sustained well inside the mid-infrared, choosing Bi_2_Te_3_ as case study within this family of materials. Strong resonant modulation of the incident electromagnetic field is achieved thanks to the exceptionally high refractive index ranging between 7 and 8 throughout the 2–10 μm region. Analysis of the complex mode structure in the metamaterial allude to the excitation of circular surface currents which could open pathways for enhanced light-matter interaction and low-loss plasmonic configurations by coupling to the spin-polarized topological surface carriers, thereby providing new opportunities to combine dielectric, plasmonic and magnetic metamaterials in a single platform.

## Abstract (web summary)

High-index dielectric materials are in great demand for nanophotonic applications. Here, the authors show that chalcogenide topological insulators are suitable candidates for dielectric nanophotonics in the infrared spectral range by reporting resonances in Bi_2_Te_3_ crystals sustained in the mid-infrared.

## Introduction

Topological insulator (TI) crystals feature time reversal symmetry-protected, highly conducting surface states characterized by Dirac dispersion and spin-momentum locking of carriers that encapsulate a semi-conducting bulk^1–3^. They are an extremely attractive class of materials for electronic^4^, spintronic^5^ and, more recently, photonic^6,7^ applications, where coupling of light to the topologically protected surface carriers may lead to propagating surface plasmon polaritons with very little scattering and other exotic phenomena, such as hybridization of spin and surface plasmons^8^. This has motivated extensive studies of electromagnetic properties of TI chalcogenide crystals over a broad range of frequencies from THz to UV^9–15^. Interaction of electromagnetic waves with the topological surface states can be enhanced by suitably structuring the TI crystals with subwavelength units, such as resonant metamolecules, giving rise to absorption and localization of the electromagnetic field^9,16^. TI metamaterials have been realized at THz and UV–visible frequencies, where the material response is more plasmonic due to free surface carriers and bound bulk carriers, respectively^9,16–18^. However, there have been hardly any studies on resonant TI structures at intermediate near-infrared and mid-infrared frequencies, where the compositionally tunable refractive index is extremely high^19^, and optical conductivity from charge carriers in topological surface states becomes significant^20^. Within the family of chalcogenide crystals, we select Bi_2_Te_3_ to demonstrate dielectric metamaterial structures in the technologically important near to mid-infrared frequency window. Bi_2_Te_3_ has a refractive index between 7 and 8 over the 2–10 μm spectral range, which is much larger than typical infrared dielectric materials, such as Si (*n* = 3.44)^21^, Ge (*n* = 4.07)^22^, PbTe (*n* = 5.61)^23^, and GST (*n* = 6–7.2)^24^.

Here we show that the high refractive index of the chalcogenide crystals can be used to generate strong infrared resonances and associated complex mode structures with surface circular currents, opening up new opportunities to couple light with spin-polarized topological surface state carriers.

## Results

### The high infrared refractive index of TIs

**Fig. 1:**
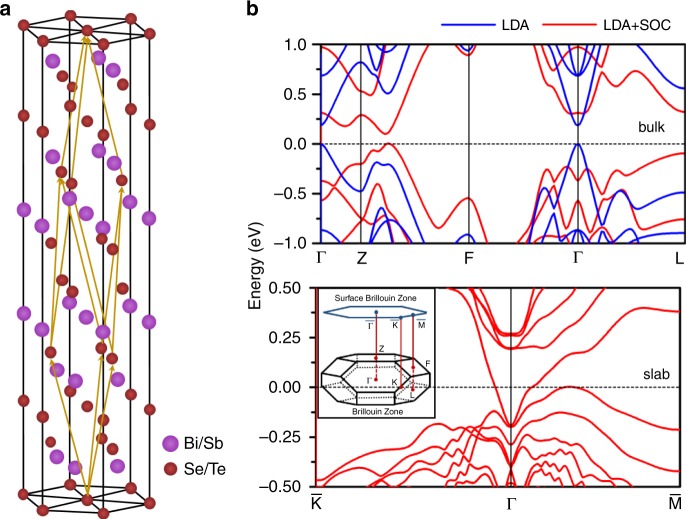
Topological insulator properties of Bi_2_Te_3_. **a** Crystal structure of rhombohedral-phase Bi_2_Te_3_. **b** Band structure of Bi_2_Te_3_ bulk (top panel) and slab (bottom panel) calculated at the LDA level, with (red lines) and without (blue lines) spin–orbit coupling. Including the latter leads to band-inversion at the Γ point and gives rise to topologically protected states at the surface (bottom panel). Inset of the bottom panel shows Brillouin zone of the crystal indicating the main symmetry points.

**Fig. 2:**
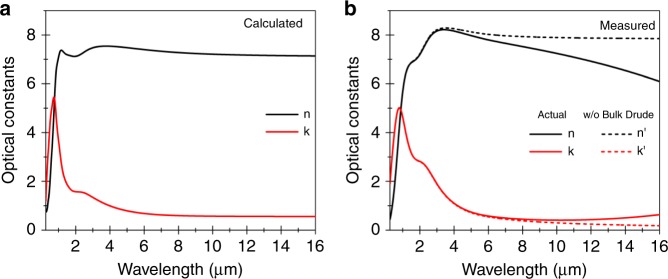
Infrared refractive index of Bi_2_Te_3_ topological insulator crystals. Dispersion of the complex refractive index **a** calculated from first-principles technique and, **b** measured experimentally (solid lines) from ellipsometry and infrared spectroscopy measurements. The dashed lines correspond to the case in which the Drude contribution from bulk free carriers is removed.

We started by carrying out first-principles calculations and spectroscopic measurements of single crystal Bi_2_Te_3_. Density functional theory (DFT) calculations based on the local density approximation (LDA) were employed to study the electronic band structures and optical properties of the rhombohedral-phase Bi_2_Te_3_ (see the crystal structure in Fig. 1a) using the Quantum ESPRESSO (QE) package^25^. Experimental lattice parameters of bulk Bi_2_Te_3_^26,27^ were used to build the initial structure, and ground states geometry of the Bi_2_Te_3_ crystals was obtained by the total energy minimization method upon relaxing their crystal framework and atomic coordinates. We calculated the band structure of Bi_2_Te_3_ with and without spin–orbit coupling (SOC). Due to the presence of heavy elements such as Bi and Te, relativistic effects and SOC have significant impact on the band structure. Without SOC, the bands have the typical parabolic dispersion with a direct gap at the Γ point. However, the presence of SOC leads to band-inversion at the Γ point and a topologically non-trivial gap is induced (Fig. 1b top panel) with the Dirac dispersion appearing in the case of a thin slab (Fig. 1b bottom panel). The optical response was calculated by employing the Bethe–Salpeter equations (BSE) method with the YAMBO code, using ground-state wavefunctions from QE package^28,29^. The imaginary part of the permittivity was determined by evaluating direct electronic transitions between occupied and higher-energy unoccupied electronic states and the real part was obtained by employing Kramers–Kroning transformation on the imaginary part. Additional details on optical response calculations can be found in ref. ^20^. Figure 2a shows the real and imaginary parts of the calculated complex refractive index ($\widetilde n = n + {\mathrm{i}}k$). Its dispersion is characterized by contrasting behaviors in two regions of the spectrum: (i) the short wavelength (0.25–1.50 μm) region with strong absorption (high *k*), resulting from interband transitions in the bulk, and (ii) the long wavelength (~2–16 μm) region featuring strong polarizability (high *n*). Correspondingly, the permittivity is negative throughout the 0.25–0.85 μm region (*n* < *k*)^9,20^, where the material is plasmonic. The typical concentration of surface carriers in these materials (10^12^–10^13^ cm^−2^) results in their contribution to the optical conductivity becoming significant in the long wavelength region above 6 μm^20^, as manifested by a small decrease of the refractive index.

Spectroscopic studies were carried out on exfoliated films of TI single crystal samples of Bi_2_Te_3_ with thickness ranging from 10 to 100 μm. We characterized optical properties over a broad spectral range, from the UV to the mid-infrared by means of variable angle ellipsometry (in the UV to near-infrared range) and near-infrared to mid-infrared reflection measurements, from which we extracted the experimental complex refractive index dispersion (Fig. 2b). The experimental dispersion compares well with the calculated one, showing absorption and negative permittivity at the shorter wavelengths, and strong dielectric behavior in the infrared.

The deviation from the calculated dispersion at longer wavelengths is due to a sharp decrease of the refractive index and an increase of the extinction coefficient induced by free bulk carriers from intrinsic doping. This effectively creates a third region of high refractive index and low losses between 7 and 10 μm for the crystals in our hands. The dashed lines in Fig. 2b show the experimental optical constants after removing the bulk Drude contribution for comparison with the DFT calculation results, showing fairly good agreement between the two once the intrinsic doping contribution is excluded.

### Broadly tunable infrared dielectric metamaterials

**Fig. 3:**
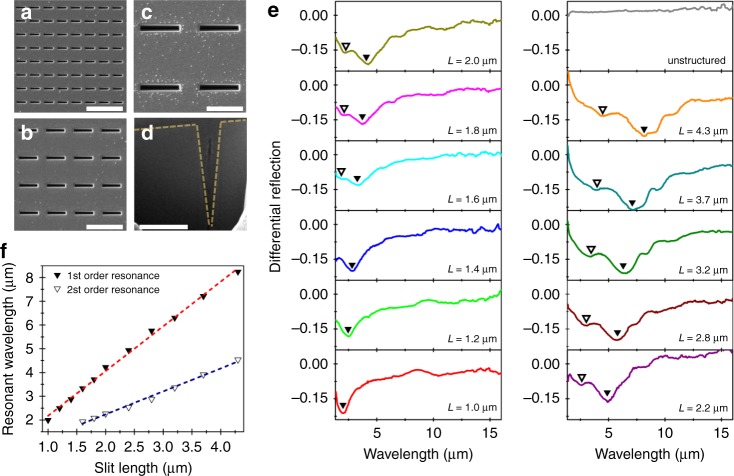
Infrared resonances of nanoslit metamaterials. **a**–**c** SEM images of slit arrays with lengths *L* = 1.0, 2.0, and 4.3 μm, respectively. Scale bar corresponds to 4 μm. **d** Cross-sectional image of slit array of length 4.3 μm. Scale bar corresponds to 2 μm. Dashed light yellow lines are an aid to mark the v-shaped contour of the slit. **e** Experimental infrared differential reflection spectra from slit arrays of different slit lengths from 1.0 to 4.3 μm. Pronounced resonances, both first (▼) and second order (▽) are observed that red-shift with the slit length. **f** Resonant wavelength as a function of slit length for the fundamental and second order resonant modes in the nanoslit arrays.

Optical materials with high refractive index and low losses such as Bi_2_Te_3_ in the mid-infrared are in great demand for dielectric metamaterials, as they can produce strong mode confinement and narrow resonances for small form factor devices^30^. On this premise, we fabricated infrared nanoslit arrays via focused ion beam milling on the surface of exfoliated Bi_2_Te_3_ crystals. The slit array geometry was chosen for geometrical simplicity and designed to have pronounced resonances across the entire infrared spectrum by varying the nanoslit length (*L*) from 1.0 to 4.3 μm. Representative top-view and cross-sectional SEM images of the fabricated nanoslit arrays are shown in Fig. 3a–d. Infrared microscopy reflection measurements were carried out with incident electric field vector polarized both parallel (${R}_{{\mathrm{E}}\parallel }$) and perpendicular (*R*_E⊥_) to the length of the slits. The slit arrays show resonant response only when excited with perpendicular polarization. To highlight these resonances, we plot the reflection spectra in a differential form, $( {R_{{\mathrm{{E}}} \bot } - R_{{\mathrm{{E}}}\parallel }} )/R_{{\mathrm{{E}}}\parallel }$, for the various slit lengths (Fig. 3e). Two distinct resonances are clearly observed in the plot, the fundamental resonances (indicated by ▼) and, the second-order resonances (indicated by ▽) which appear in the measured spectral region for slits longer than 1.5 μm. Figure 3f shows the variation of the resonant wavelength as a function of slit length, clearly indicating a linear spectral red-shift with scaling factors of 2 and 1 times the slit length for the fundamental and second-order resonances, respectively^31^.

### Nature of resonant modes and circular surface currents

To further our understanding of the nature of the main resonant mode, we carried out finite-element method (FEM) simulations of the nanoslit array, over the corresponding wavelength interval.

**Fig. 4:**
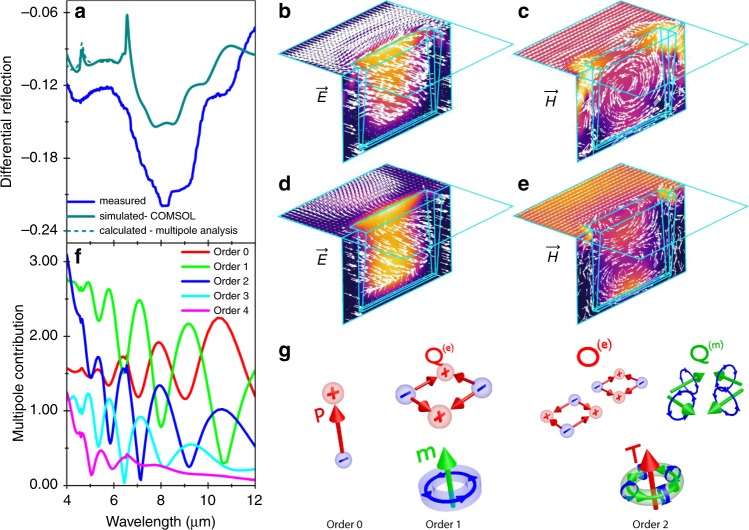
Field maps and complex mode structure. **a** Experimental (solid blue), simulated (solid dark cyan) and calculated (dashed lines) differential reflection spectra of Bi_2_Te_3_ metamaterial slit array of length 4.30 μm. The fundamental and second-order resonances at 8.55 and 4.35 μm, respectively, are evident in the plot. The sharp peaks in the simulated and calculated spectra around 4.60 and 6.50 μm are due to diffraction effects. Maps of electric (E) and, magnetic (H) fields determined by FEM simulations showing the nature of the mode at the, **b**, **c** fundamental, and **d**, **e** second-order resonances. The maps have been calculated over one half of the slit, for simulation size purposes. The cyan outline overlaid on the color maps indicates the full slit and simulation region for clarity. **f** The contribution of various multipoles to the (displacement) current excitation induced in the metamaterial by the incident wave polarized perpendicular to the slits, including electric dipole (order 0), magnetic dipole (order 1), electric quadrupole (order 1), and toroidal dipole (order 2). **g** Schematic representation of the various modes in the metamaterial. See Supplementary Note 1 for further details.

Figure 4a compares the experimental and simulated differential reflection spectra of the longest slit (*L* = 4.3 μm) arrays, showing good agreement between the two. Corresponding maps of the resonant electric and magnetic fields at 8.55 μm are plotted in Fig. 4b, c for incident electric field polarization perpendicular to the slits. The field-maps for the minor mode at 4.35 μm are plotted in Fig. 4d, e. Contrary to conventional expectations, a circulating pattern for the magnetic field as displayed by the mode at 8.55 μm (Fig. 4b, c), does not necessarily imply that the resonance is a toroidal dipole. The exact multipole decomposition, shown in Fig. 4f, shows that the situation is far more nuanced: the plot of the multipole components expressed in terms of the current density (see Supplementary Eq. (12)) shows that the metamaterial excitation is not dominated by a particular mode, but is a combination of multiple modes, such as the electric dipole (order 0), magnetic dipole and electric quadrupole (order 1), toroidal dipole, magnetic dipole, and electric quadrupole (order 2), etc., depicted schematically in Fig. 4g. Noteworthy is the fact that toroidal-like field configurations can be generated in the presence of multiple modes. The differential reflection spectrum calculated from multipole analysis is shown as dashed lines in Fig. 4a and agrees fairly well with the simulated and experimental spectra. For more details of the multipole analysis, refer to Supplementary Note 1. The multipole contribution for incident non-resonant polarization (electric field parallel to the slit length) is shown in Supplementary Fig. 2. We note that the overall reflection response of the slit arrays depends on the interference of the various multipoles based on their amplitude and phases which is rather complicated and can be illustrated with the aid of phasor diagrams (Supplementary Note 3). We also note that the minor mode at 4.35 μm also shows similar behavior with circulating magnetic fields but with two vortices indicating that it is a second-order mode. Supplementary Movies 1–4 show the evolution of the electric and magnetic fields within the metamaterial over half a cycle (0–*π*) of the phase.

## Discussion

The high-order modes supported by metamaterials^32–34^ enable strong light localization and confinement, which may be suitable for applications in nonlinear and laser optics^35,36^, or in coupling to high-order transitions in atoms or molecules. In the context of TI metamaterials, the unique nature of the resonant fields featuring circular currents on the surface of the TI may be exploited to couple light with spin-polarized carriers and gain optical access to the topological surface states that encapsulate the material conformally^37^. This becomes particularly relevant in the mid-IR region, where chalcogenide crystals feature a combination of high refractive index and larger contribution of topological surface states to the optical conductivity.

Overall, the TI chalcogenide crystal family is an exceptionally versatile material platform for infrared applications based on high-index, low-loss dielectric metamaterial architectures^38^, including ultrathin flat optical elements^39^, sub-diffraction light confinement and waveguiding^40^, and nonlinear optics^41^. Low-loss mid-IR metamaterials are also highly sought for enhanced sensing of molecular fingerprints based on strong light confinement^42,43^. Chalcogenide crystal metamaterials add broadband tunability of the resonances by compositional^44^ and structural design to the high refractive index. Moreover, as shown here for Bi_2_Te_3_, the inverse geometry of nanoslits carved in high-index crystals induces higher-order complex modes outside the dielectric medium, which provide additional pathways to sense changes in the surrounding environment.

In conclusion, chalcogenide TI crystals are a compelling materials platform for photonic applications in the infrared part of the spectrum. We have shown that Bi_2_Te_3_ exhibits a strong polarizability with refractive index that exceeds 7 in the 2–10 μm range, larger than conventional dielectric materials that aids strong nanostructure resonances sustained deep into the mid-infrared. The exceptionally high index facilitates formation of circular currents at the surface of the material that may potentially be coupled to spin-polarized topological surface states. This opens the path to new infrared metamaterials combining dielectric, plasmonic, and magnetic properties for applications including molecular fingerprinting, environmental sensing, and integrated mid-IR photonics.

## Methods

### Optical characterization

Bi_2_Te_3_ crystals were purchased commercially from 2D Semiconductors Inc. The infrared reflection/transmission spectra of the unstructured TIs crystals and microscopy reflection spectra of structured Bi_2_Te_3_ were measured using a Bruker Hyperion microscope coupled to a Bruker Vertex 80v spectrometer. Spectroscopic ellipsometry data were collected using a J.A. Woollam VASE ellipsometer in the 250–1650 nm spectral range over three angles of incidence (30°, 50°, and 70°), and analyzed using the CompleteEASE ellipsometry data analysis program. The refractive index values in the infrared were determined using the experimentally measured near-to-mid-infrared reflection spectrum and the dielectric constants in the UV–near infrared range measured from ellipsometry. This analysis was carried out using the RefFIT program wherein a combination of Tauc–Lorentz, Lorentz, and Drude oscillators were used to model simultaneously, the ellipsometric dielectric constants as well as the infrared reflection spectrum, from which the refractive index of the material in the infrared spectral range was determined. In the Reffit program, the infrared reflection response was modeled using the model ‘Reflectivity of a Film (Epsilon + Mu) on a Substrate (code = −18)’. The parameters of the oscillators describing the material optical function are shown in Table 1.

**Table 1:** Optical response function model.

Oscillator	Formula	Fitted parameters (cm^−1^)
Tauc–Lorentz	$\varepsilon _2 = \frac{1}{\omega }\frac{{A\omega _0\gamma \left( {\omega - \omega _{\mathrm{{g}}}} \right)^2}}{{\left( {\omega ^2 - \omega _0^2} \right)^2}}{\mathrm{\Theta }}\left( {\omega - \omega _{\mathrm{{g}}}} \right)$ $\varepsilon _1 = 1 + \frac{2}{\pi }\mathop {\int}\limits_0^\infty {\frac{{\omega ^{\prime} \varepsilon _2\left( {\omega ^{\prime} } \right){\mathrm{{d}}}\omega ^{\prime} }}{{\omega ^{\prime 2} - \omega ^2}}}$	*ω*_0_ = 10,902 *ω*_g_ = 1300 *A* = 7.69e^5^ *γ* = 12,229
Lorentz	$\varepsilon \left( \omega \right) = \varepsilon _\infty + \frac{{\omega _{\mathrm{{p}}}^2}}{{\omega _0^2 - \omega ^2 - i\gamma \omega }}$	$\varepsilon _\infty = 1$; *ω*_0_ = 4343.7 *ω*_p_ = 19,088; *γ* = 3625.4
Drude	$\varepsilon \left( \omega \right) = \varepsilon _\infty + \frac{{\omega _{\mathrm{{p}}}^2}}{{\omega _0^2 - \omega ^2 - i\gamma \omega }}$	$\varepsilon _\infty = 1$; *ω*_0_ = 0 *ω*_p_ = 3147.5; *γ* = 165.6

Parameters of the oscillators used to model the dielectric function of Bi_2_Te_3_ crystals throughout the optical spectral range.

### Simulations

The optical response of the nanoslit array was simulated using full-wave Maxwell equations solver COMSOL^45^. The simulations were carried out for a 3D structure using perfect electric/magnetic conductor boundary conditions on the mirror symmetry plane containing the long axis of the slit for electric field polarized perpendicular/parallel to the slit. In the multipole analysis, the number of higher-order modes obtained is dependent on the position of the air–metamaterial interface relative to the *z* = 0 plane. The optimal position of this interface is chosen such that the differential reflection as well as the field induced in the metamaterial can be quantified with the minimum possible number of modes (in this case 5), and corresponds to z = −1200 nm for the mode contributions shown in Fig. 4f.

## Supplementary information

Supplementary Information

Supplementary Movie 1

Supplementary Movie 2

Supplementary Movie 3

Supplementary Movie 4

Description of Additional Supplementary Files

## Data Availability

Following a period of embargo, the data from this paper can be obtained from the University of Southampton ePrints research repository, 10.5258/SOTON/D1252.

## Code availability

Following a period of embargo, the codes used in this paper can be obtained from the University of Southampton ePrints research repository, 10.5258/SOTON/D1252.

## References

[1] Moore JE (2010). The birth of topological insulators. Nature.

[2] Hasan MZ, Kane CL (2010). *Colloquium*: topological insulators. Rev. Mod. Phys..

[3] Ando Y (2013). Topological insulator materials. J. Phys. Soc. Jpn..

[4] McIver JW, Hsieh D, Steinberg H, Jarillo-Herrero P, Gedik N (2012). Control over topological insulator photocurrents with light polarization. Nat. Nanotechnol..

[5] Pesin D, MacDonald AH (2012). Spintronics and pseudospintronics in graphene and topological insulators. Nat. Mater..

[6] Graydon O (2017). A question of topology. Nat. Photonics.

[7] Yue Z, Cai B, Wang L, Wang X, Gu M (2016). Intrinsically core–shell plasmonic dielectric nanostructures with ultrahigh refractive index. Sci. Adv..

[8] Stockman MI (2018). Roadmap on plasmonics. J. Opt..

[9] Ou J-Y (2014). Ultraviolet and visible range plasmonics in the topological insulator Bi_1.5_Sb_0.5_Te_1.8_Se_1.2_. Nat. Commun..

[10] Tang CS (2013). Terahertz conductivity of topological surface states in Bi_1.5_Sb_0.5_Te_1.8_ Se_1.2_. Sci. Rep..

[11] Zhao M (2015). Visible surface plasmon modes in single Bi_2_Te_3_ nanoplate. Nano Lett..

[12] Whitney WS (2017). Gate-variable mid-infrared optical transitions in a (Bi_1– *x*_Sb_*x*_)_2_Te_3_ topological insulator. Nano Lett..

[13] Esslinger M (2014). Tetradymites as natural hyperbolic materials for the near-infrared to visible. ACS Photonics.

[14] Dubroka A (2017). Interband absorption edge in the topological insulators Bi_2_(Te_1-x_Se_x_)_3_. Phys. Rev. B.

[15] Post KW (2015). Infrared probe of the bulk insulating response in Bi_2-x_Sb_x_Te_3-y_Se_y_ topological insulator alloys. Phys. Rev. B.

[16] Di Pietro P (2013). Observation of Dirac plasmons in a topological insulator. Nat. Nanotechnol..

[17] Autore M (2015). Plasmon–phonon interactions in topological insulator microrings. Adv. Opt. Mater..

[18] Dubrovkin AM (2017). Visible range plasmonic modes on topological insulator nanostructures. Adv. Opt. Mater..

[19] Piccinotti, D. et al. Stoichiometric engineering of chalcogenide semiconductor alloys for nanophotonic applications. *Adv. Mater*. 1807083 (2019). 10.1002/adma.201807083 10.1002/adma.201807083 30773719

[20] Yin J (2017). Plasmonics of topological insulators at optical frequencies. NPG Asia Mater..

[21] Chandler-Horowitz D, Amirtharaj PM (2005). High-accuracy, midinfrared (450 cm^−1 ^⩽ ω ⩽ 4000 cm^−1^) refractive index values of silicon. J. Appl. Phys..

[22] Jellison GE (1992). Optical functions of GaAs, GaP, and Ge determined by two-channel polarization modulation ellipsometry. Opt. Mater..

[23] Weiting F, Yixun Y (1990). Temperature effects on the refractive index of lead telluride and zinc selenide. Infrared Phys..

[24] Michel A-KU, Wuttig M, Taubner T (2017). Design parameters for phase-change materials for nanostructure resonance tuning. Adv. Opt. Mater..

[25] Giannozzi P (2009). QUANTUM ESPRESSO: a modular and open-source software project for quantum simulations of materials. J. Phys. Condens. Matter.

[26] *Non-Tetrahedrally Bonded Elements and Binary Compounds I,* Vol. 41C (Springer-Verlag, 1998).

[27] Ren Z, Taskin AA, Sasaki S, Segawa K, Ando Y (2011). Optimizing Bi_2-x_Sb_x_Te_3−y_Se_y_ solid solutions to approach the intrinsic topological insulator regime. Phys. Rev. B.

[28] Marini A, Hogan C, Grüning M, Varsano D (2009). yambo: an ab initio tool for excited state calculations. Comput. Phys. Commun..

[29] Rohlfing M, Louie SG (2000). Electron–hole excitations and optical spectra from first principles. Phys. Rev. B.

[30] Baranov DG (2017). All-dielectric nanophotonics: the quest for better materials and fabrication techniques. Optica.

[31] Aizpurua J (2005). Optical properties of coupled metallic nanorods for field-enhanced spectroscopy. Phys. Rev. B.

[32] Savinov V, Fedotov VA, Zheludev NI (2014). Toroidal dipolar excitation and macroscopic electromagnetic properties of metamaterials. Phys. Rev. B.

[33] Papasimakis N, Fedotov VA, Savinov V, Raybould TA, Zheludev NI (2016). Electromagnetic toroidal excitations in matter and free space. Nat. Mater..

[34] Tian J (2019). Active control of anapole states by structuring the phase-change alloy Ge_2_Sb_2_Te_5_. Nat. Commun..

[35] Baryshnikova KV, Smirnova DA, Luk’yanchuk BS, Kivshar YS (2019). Optical anapoles: concepts and applications. Adv. Opt. Mater..

[36] Grinblat G, Li Y, Nielsen MP, Oulton RF, Maier SA (2016). Enhanced third harmonic generation in single germanium nanodisks excited at the anapole mode. Nano Lett..

[37] Tokura Y, Yasuda K, Tsukazaki A (2019). Magnetic topological insulators. Nat. Rev. Phys..

[38] Verre R (2019). Transition metal dichalcogenide nanodisks as high-index dielectric Mie nanoresonators. Nat. Nanotechnol..

[39] Zhang L (2018). Ultra-thin high-efficiency mid-infrared transmissive Huygens meta-optics. Nat. Commun..

[40] Jin T, Zhou J, Lin H-YG, Lin PT (2019). Mid-infrared chalcogenide waveguides for real-time and nondestructive volatile organic compound detection. Anal. Chem..

[41] Xu Y (2018). Reconfiguring structured light beams using nonlinear metasurfaces. Opt. Express.

[42] Tittl A (2018). Imaging-based molecular barcoding with pixelated dielectric metasurfaces. Science.

[43] Leitis A (2019). Angle-multiplexed all-dielectric metasurfaces for broadband molecular fingerprint retrieval. Sci. Adv..

[44] Gholipour B, Piccinotti D, Karvounis A, MacDonald KF, Zheludev NI (2019). Reconfigurable ultraviolet and high-energy visible dielectric metamaterials. Nano Lett..

[45] COMSOL Multiphysics Reference Manual, version 5.3. COMSOL, Inc, www.comsol.com.

