# Supplementary material for: Infrared dielectric metamaterials from high refractive index chalcogenides
Source: Nat Commun. 2020 Apr 3;11:1692. doi: 10.1038/s41467-020-15444-0 (PMC7125163; doi:10.1038/s41467-020-15444-0)
Supplement: Supplementary file 1 — Supplementary Information [file 41467_2020_15444_MOESM1_ESM.pdf]

Supplementary Information
Infrared dielectric metamaterials from high refractive index Bi_2Te_3 crystals

Krishnamoorthy, Adamo et al.

SUPPLEMENTARY NOTE 1: MULTIPOLE DECOMPOSITION FOR SANDWICHED META-SURFACES

The aim of this section is to establish the multipole decomposition of electromagnetic excitations induced in planar metamaterials, i.e. meta-surfaces, sandwiched between two semi-infinite media with different refractive indices.

Conventional approach is unsuitable

Conventional approach to multipole decomposition of meta-surface excitations involves integrating over the current density within the unit cell of the metamaterial [1]. The periodicity of the meta-surface limits the in-plane size of the unit cell. The out-of-plane size of the unit cell, i.e. the size of the unit cell along the direction perpendicular to metamaterial plane, is conventionally limited by defining the current density in such a way that it vanishes identically in the ambient environment. For example, if the meta-surface is placed into the environment with ambient index of refraction n_{amb}. One can define the current density to be[2]:

$$\mathbf{J}\left(\mathbf{r}\right) = i\omega\epsilon_0 \left(n\left(\mathbf{r}\right)^2 - n_{amb}^2\right)\mathbf{E}\left(\mathbf{r}\right)$$

Where ω is the angular frequency, ϵ_0 is the vacuum permittivity, $n\left(\mathbf{r}\right)$ is the position-dependent complex refractive index, and $\mathbf{E}$ is the local electric field. Such definition ensures that current density, and therefore the multipole contribution of electromagnetic fields outside the metamaterials will be identically zero.

Clearly, such approach cannot work if there are two different ambient media either side of the meta-surface as shown in Fig. 1.

Domain merging

Consider a vertical meta-surface sandwiched between an ambient medium with refractive index n_1, on the left-hand-side, and a substrate with refractive index n_s, on the right-hand side. The meta-surface is driven by normally-incident plane wave radiation traveling from left to right, as shown in Fig. 1.

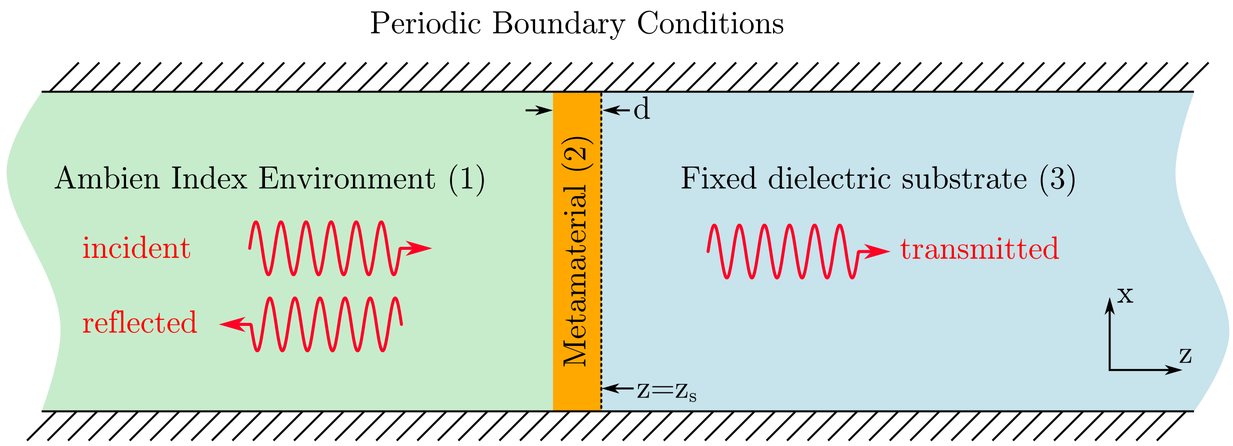

Supplementary Figure 1. The setup for the domain merging treatment. A plane wave is incident onto the metamaterial (meta-surface) from half-space filled with ambient dielectric material (domain 1). The metamaterial is treated as a homogeneous layer (domain 2). The light is reflected into the ambient index medium and transmitted into the dielectric substrate (domain 3). Metamaterial slab thickness is d. The position of the metamaterial-substrate interface is $z = z_s$.

Here the metamaterial is treated as a slab that may change along z-axis, but is homogeneous as far as xy-translations are concerned. This approach has been shown to be valid even in case of non-homogeneous unit-cells, e.g. split-ring resonator metamaterials, as long as one considers normal incidence and diffraction can be ignored [1]. The metamaterial-substrate boundary is at $z = z_s$, the metamaterial-ambient environment boundary is at $z = z_s - d$.

The electric field in all space is subject to equation:

$$\left(\nabla^2 + k^2\right)\boldsymbol{E} = \boldsymbol{0} \tag{1}$$

Where k is the local wave-number which will depend on the medium at the chosen location. We shall proceed by working with two wave equations: one for domains 1&2, another for domain 3. In essence, the treatment revolves around merging domains 1 and 2 into a single domain with added 'current density'.

In domains 1&2 the wave equation can be transformed as follows[3]:

$$\begin{gathered}\left(\partial_z^2 + k^2\right)\boldsymbol{E} = \boldsymbol{0}\\ \left(\partial_z^2 + k_1^2\right)\boldsymbol{E} + \left(k^2 - k_1^2\right)\boldsymbol{E} = \boldsymbol{0}\\ \left(\partial_z^2 + k_1^2\right)\boldsymbol{E} = -\boldsymbol{\Upsilon}\\ \boldsymbol{\Upsilon} = \left(k^2 - k_1^2\right)\boldsymbol{E}\end{gathered}$$

Where k_1 is the wave-number in domain 1. Now $\boldsymbol{\Upsilon}$ can be treated as a 'source' in the domain 1&2. Formally, the solution to this equation will be:

$$\begin{gathered}\boldsymbol{E} = \boldsymbol{E}_{hom} + \frac{-i}{2k_1}\int_{-\infty}^{z_s} dz'\,\boldsymbol{\Upsilon}\left(z'\right)\exp\left(-ik_1\left|z - z'\right|\right)\\ \left(\partial_z^2 + k_1^2\right)\boldsymbol{E}_{hom} = \boldsymbol{0}\end{gathered}$$

In anticipation of what is to come, one can set the homogeneous solution to accommodate transmission and reflection, i.e. scattering boundary conditions with incident wave of amplitude$\mathbf{E}_i$ and propagating along the positive-z direction. It is convenient to parametrize the vectors in the metamaterial plane in terms of vectors $\hat{\mathbf{u}} \parallel \mathbf{E}_i$ and $\hat{\boldsymbol{u}} \times \hat{\boldsymbol{v}} = \hat{\boldsymbol{z}}$:

$$\begin{aligned}\boldsymbol{E}_{1\&2} =& \hat{\boldsymbol{u}}E_i\exp\left(-ik_1z\right) + \hat{\boldsymbol{u}}\rho_{uu}E_i\exp\left(ik_1z\right) + \hat{\boldsymbol{v}}\rho_{vu}E_i\exp\left(ik_1z\right) + \\ &+ \left(\frac{-i}{2k_1}\right)\int_{-\infty}^{z_s} dz'\,\boldsymbol{\Upsilon}\left(z'\right)\exp\left(-ik_1\left|z - z'\right|\right)\end{aligned} \tag{2}$$

$$\begin{aligned}\boldsymbol{H}_{1\&2} =& \hat{\boldsymbol{v}}H_i\exp\left(-ik_1z\right) - \hat{\boldsymbol{v}}\rho_{uu}H_i\exp\left(ik_1z\right) + \hat{\boldsymbol{u}}\rho_{vu}H_i\exp\left(ik_1z\right) + \\ &+ \left(\frac{-i}{2\omega\mu_0}\right)\int_{-\infty}^{z_s} dz'\,\left(\hat{\boldsymbol{z}} \times \boldsymbol{\Upsilon}\left(z'\right)\right)\exp\left(-ik_1\left|z - z'\right|\right)\cdot\operatorname{sign}\left(z - z'\right)\end{aligned} \tag{3}$$

Where $\boldsymbol{\rho}$ is the complex-valued matrix related to reflection[4], and $H_i = k_1E_i/\omega\mu_0$ is the corresponding amplitude of the magnetic field (μ_0 is the vacuum permeability). The sign-function above vanishes at zero ($\operatorname{sign}(0) \equiv 0$).

Next the solution in domain 3:

$$\boldsymbol{E}_3 = \hat{\boldsymbol{u}}t_{uu}E_i\exp\left(-ik_sz\right) + \hat{\boldsymbol{v}}t_{vu}E_i\exp\left(-ik_sz\right) \tag{4}$$

$$\boldsymbol{H}_3 = \hat{\boldsymbol{v}}t_{uu}\frac{k_s}{k_1}H_i\exp\left(-ik_sz\right) - \hat{\boldsymbol{u}}t_{vu}\frac{k_s}{k_1}H_i\exp\left(-ik_sz\right) \tag{5}$$

Where $\boldsymbol{t}$ is the transmission matrix and k_s is the wave-number in the substrate. Now one needs the boundary conditions to stitch up the domains 1&2 and 3. Assuming one only has to worry about the transverse fields (those are the only ones that will propagate out), one simply has to ensure the continuity of electric and magnetic fields at $z = z_s$:

$$\exp\left(-ik_1z_s\right) + \rho_{uu}\exp\left(ik_1z_s\right) + \left(\frac{-i}{2k_1}\right)\int_{-\infty}^{z_s} dz'\,k_0^2\bar{\Upsilon}_u\left(z'\right)\exp\left(ik_1z'\right)\exp\left(-ik_1z_s\right) = t_{uu}\exp\left(-ik_sz_s\right) \tag{6}$$

$$\rho_{vu}\exp\left(ik_1z_s\right) + \left(\frac{-i}{2k_1}\right)\int_{-\infty}^{z_s} dz'\,k_0^2\bar{\Upsilon}_v\left(z'\right)\exp\left(ik_1z'\right)\exp\left(-ik_1z_s\right) = t_{vu}\exp\left(-ik_sz_s\right) \tag{7}$$

$$\exp\left(-ik_1z_s\right) - \rho_{uu}\exp\left(ik_1z_s\right) + \left(\frac{-i}{2k_1}\right)\int_{-\infty}^{z_s} dz'\,k_0^2\bar{\Upsilon}_u\left(z'\right)\exp\left(ik_1z'\right)\exp\left(-ik_1z_s\right) = t_{uu}\frac{k_s}{k_1}\exp\left(-ik_sz_s\right) \tag{8}$$

$$\rho_{vu}\exp\left(ik_1z_s\right) + \left(\frac{-i}{2k_1}\right)\int_{-\infty}^{z_s} dz'\,k_0^2\left(-\bar{\Upsilon}_v\left(z'\right)\right)\exp\left(ik_1z'\right)\exp\left(-ik_1z_s\right) = -t_{vu}\frac{k_s}{k_1}\exp\left(-ik_sz_s\right) \tag{9}$$

Where $\mathbf{\Upsilon} = k_0^2 E_i \bar{\mathbf{\Upsilon}}$. Note that at the $z = z_s$ boundary we have $z \geq z'$, thus we must evaluate $|z - z'| = z - z'$, however for the actual evaluation of reflection, we will have $z \ll z'$, thus $|z - z'| \to z' - z$.

To keep things tractable, we will assume that there is no polarization conversion, i.e. $t_{vu} = t_{uv} = 0$. In this case:

$$\rho_{vu} \exp(ik_1 z_s) + \left(\frac{-i}{2k_1}\right) \int_{-\infty}^{z_s} dz'\, k_0^2 \bar{\Upsilon}_v(z') \exp(ik_1 z') \exp(-ik_1 z_s) = 0$$

But due to linear independence of complex exponentials this only works for $\bar{\Upsilon}_v = 0$ and $\rho_{vu} = 0$. Let $\bar{\Upsilon} \equiv \bar{\Upsilon}_u$ and $\rho \equiv \rho_{uu}$ and same for t:

$$\exp(-ik_1 z_s) + \rho \exp(ik_1 z_s) + \left(\frac{-i}{2k_1}\right) \int_{-\infty}^{z_s} dz'\, k_0^2 \bar{\Upsilon}(z') \exp(ik_1 z') \exp(-ik_1 z_s) = t \exp(-ik_s z_s) \tag{10}$$

$$\exp(-ik_1 z_s) - \rho \exp(ik_1 z_s) + \left(\frac{-i}{2k_1}\right) \int_{-\infty}^{z_s} dz'\, k_0^2 \bar{\Upsilon}(z') \exp(ik_1 z') \exp(-ik_1 z_s) = t \frac{k_s}{k_1} \exp(-ik_s z_s) \tag{11}$$

Next, we expand the complex exponential $\exp(ik_1 z')$, and introduce[5]:

$$\frac{\mathcal{M}^{(l)}}{\Delta^2} = \int_{-\infty}^{z_s} (dz'\, k_0)\ \bar{\Upsilon}(z')\ (k_0 z')^l$$

$$k_0 \sum_{l=0}^{\infty} \frac{(in_1)^l}{l!} \frac{\mathcal{M}^{(l)}}{\Delta^2} = \int_{-\infty}^{z_s} dz'\, k_0^2 \bar{\Upsilon}(z') \exp(ik_1 z')$$

$$\mathcal{M}^{(l)} = \int_u d^3r'\, k_0\ (k_0 z')^l\ \bar{\Upsilon}(\mathbf{r}') = \frac{k_0^{1+l}}{E_i} \int_u d^3r\, z^l\ \left(n(\mathbf{r})^2 - n_1^2\right) E_u(\mathbf{r})$$

Where Δ^2 is the area of the unit cell of the metamaterial and $\int_u d^3r'$ is the integral over the unit cell of metamaterial, since for a homogeneous metamaterial $\int_{-\infty}^{z_s} (dz'\, k_0)\ \bar{\Upsilon}(z')\ (k_0 z')^l = \frac{1}{\Delta^2} \int_u d^3r'\, k_0\ \bar{\Upsilon}(z')\ (k_0 z')^l$. Note that $\mathcal{M}^{(l)}/\Delta^2$ is unit-less. We can also express it in terms of the current density in the material:

$$\mathbf{J} = i\omega\epsilon_0 \left(n^2 - n_1^2\right) \mathbf{E} = ik_0 \mu_0 / c \left(n^2 - n_1^2\right) \mathbf{E}$$

$$\mathcal{M}^{(l)} = \frac{-ik_0^l}{E_i \epsilon_0 c} \int_u d^3r\, z^l\ J_u(\mathbf{r}) \tag{12}$$

Such current density definition agrees with the conventional definition from Sec. . The two equations then become:

$$\exp(-ik_1 z_s) + \rho \exp(ik_1 z_s) + \exp(-ik_1 z_s) \cdot \left(\frac{-i}{2n_1}\right) \sum_{l=0}^{\infty} \frac{(in_1)^l}{l!} \frac{\mathcal{M}^{(l)}}{\Delta^2} = t \exp(-ik_s z_s) \tag{13}$$

$$\exp(-ik_1 z_s) - \rho \exp(ik_1 z_s) + \exp(-ik_1 z_s) \cdot \left(\frac{-i}{2n_1}\right) \sum_{l=0}^{\infty} \frac{(in_1)^l}{l!} \frac{\mathcal{M}^{(l)}}{\Delta^2} = t \frac{n_s}{n_1} \exp(-ik_s z_s) \tag{14}$$

Adding and simplifying we get the transmission:

$$t = \exp(-ik_0 (n_1 - n_s) z_s) \cdot \frac{2n_1}{n_1 + n_s} \cdot \left(1 - \frac{i}{2n_1} \sum_{l=0}^{\infty} \frac{(in_1)^l}{l!} \cdot \frac{\mathcal{M}^{(l)}}{\Delta^2}\right) \tag{15}$$

Equivalently, subtracting we get:

$$\rho = \frac{1}{2} \cdot \exp(-i(k_1 + k_s) z_s) \cdot t \cdot \left(\frac{n_1 - n_s}{n_1}\right) \tag{16}$$

$$\rho = \exp(-i2k_1 z_s) \cdot \left(\frac{n_1 - n_s}{n_1 + n_s}\right) \cdot \left(1 - \frac{i}{2n_1} \sum_{l=0}^{\infty} \frac{(in_1)^l}{l!} \cdot \frac{\mathcal{M}^{(l)}}{\Delta^2}\right) \tag{17}$$

This, however is not the full reflection. Indeed, the solution for the domain 1&2 is:

$$\boldsymbol{E}_{1\&2} = \hat{\boldsymbol{u}} E_i \exp(-ik_1 z) + \hat{\boldsymbol{u}} \rho_{uu} E_i \exp(ik_1 z) + \hat{\boldsymbol{v}} \rho_{vu} E_i \exp(ik_1 z) +$$
$$+ \left(\frac{-i}{2k_1}\right) \int_{-\infty}^{z_s} dz'\, \mathbf{\Upsilon}(z') \exp(-ik_1 |z - z'|)$$

Dropping polarization conversion, and taking $z \to -\infty$, so that $|z - z'| = z' - z$ one finds:

$$\begin{aligned} E_{1\&2,u} =& E_i \exp\left(-ik_1 z\right) + \rho E_i \exp\left(ik_1 z\right) + \\ &+ \exp\left(ik_1 z\right) \cdot \left(\frac{-i}{2k_1}\right) \int_{-\infty}^{z_s} dz' \, E_i k_0^2 \bar{\Upsilon}\left(z'\right) \exp\left(-ik_1 z'\right) \end{aligned}$$

So the reflection coefficient is:

$$\begin{aligned} r =& \rho + \left(\frac{-i}{2k_1}\right) \int_{-\infty}^{z_s} dz' \, k_0^2 \bar{\Upsilon}\left(z'\right) \exp\left(-ik_1 z'\right) \\ =& \rho + \left(\frac{-i}{2n_1}\right) \int_{-\infty}^{z_s} dz' \, k_0 \cdot \bar{\Upsilon}\left(z'\right) \exp\left(-ik_1 z'\right) \\ =& \rho - \frac{i}{2n_1} \sum_{l=0}^{\infty} \frac{\left(-in_1\right)^l}{l!} \cdot \int_{-\infty}^{z_s} dz' \, k_0 \cdot \bar{\Upsilon}\left(z'\right) \cdot \left(k_0 z'\right)^l \\ =& \rho - \frac{i}{2n_1} \sum_{l=0}^{\infty} \frac{\left(-in_1\right)^l}{l!} \cdot \frac{\mathcal{M}^{(l)}}{\Delta^2} \end{aligned}$$

Substituting the expression for ρ:

$$r = \exp\left(-i2k_1 z_s\right) \cdot \left(\frac{n_1 - n_s}{n_1 + n_s}\right) \cdot \left(1 - \frac{i}{2n_1} \sum_{l=0}^{\infty} \frac{\left(in_1\right)^l}{l!} \cdot \frac{\mathcal{M}^{(l)}}{\Delta^2}\right) - \frac{i}{2n_1} \sum_{l=0}^{\infty} \frac{\left(-in_1\right)^l}{l!} \cdot \frac{\mathcal{M}^{(l)}}{\Delta^2}$$

Finally:

$$r = \exp\left(-i2k_0 n_1 z_s\right) \cdot \left(\frac{n_1 - n_s}{n_1 + n_s}\right) - \frac{i}{2n_1} \cdot \sum_{l=0}^{\infty} \frac{1}{l!} \cdot \left(\exp\left(-i2k_0 n_1 z_s\right) \cdot \left(\frac{n_1 - n_s}{n_1 + n_s}\right) \cdot \left(in_1\right)^l + \left(-in_1\right)^l\right) \cdot \frac{\mathcal{M}^{(l)}}{\Delta^2} \quad (18)$$

Relationship of $\mathcal{M}^{(l)}$ to multipoles

The initial motivation for this section was to find a well-behaved multipole decomposition for the metamaterial sandwiched between two different dielectric media. The resultant expressions for the transmission and reflection, however, are given in terms of $\mathcal{M}^{(l)}$ coefficients. In this sub-section we define a link between the more conventional multipoles and the introduced $\mathcal{M}^{(l)}$ coefficients.

The $\mathcal{M}^{(l)}$, as defined in Eq. (12), can be seen as a single normalized component of a more general tensor:

$$K^{\alpha_1 \ldots \alpha_l \beta}\left(t\right) = \int_u d^3 r \, r^{\alpha_1} \ldots r^{\alpha_l} \, J^{\beta}\left(\mathbf{r}, t\right)$$

obtained by setting $\alpha_1 = \alpha_2 = \cdots = \alpha_l = 3$ and $\beta = u$. The conventional multipoles can be seen as irreducible components of $K^{\alpha_1 \ldots \alpha_l \beta}$, that correspond to irreducible representations of the rotations group $SO(3)$. Including the space-inversions and time-reversal operations, introduces several types of multipoles: electric, magnetic and toroidal multipoles, in particular [6, 7]. It follows that all components of $K^{\alpha_1 \ldots \alpha_l \beta}$ can be expressed in terms of multipole components.

For example the $l = 0$ term corresponds to electric dipole ($\mathbf{d}$) along the $\hat{\mathbf{u}}$:

$$i\omega \hat{\mathbf{u}}.\mathbf{d} = \int d^3 r \, J_u$$

Here we are using the multipole definitions from Refs. [1, 8].

The $l = 1$ term corresponds to a super-position of a magnetic dipole $\mathbf{m}$ and electric quadrupole $\mathbf{Q}^{(e)}$:

$$c\hat{\mathbf{v}}.\mathbf{m} + i\omega \hat{\mathbf{u}}.\mathbf{Q}^{(e)}.\hat{\mathbf{z}} = \int d^3 r \, z J_u$$

Where c is the speed of light in the vacuum. We also state, without proof, that the $l = 2$ term corresponds to electric octupole, magnetic quadrupole and toroidal dipole. The general trend of needing superpositions of ever

growing number of multipole terms to capture $\mathcal{M}^{(l)}$ continues as l increases. It therefore becomes unpracticale to keep breaking the metamaterial excitations into multipole terms. Instead, it is far simpler to work with $\mathcal{M}^{(l)}$ terms without unpacking them.

The multipole orders in Fig. 4d, in the main paper are plots of $\mathcal{M}^{(l)}/l!$ for the l-th order of multipoles. We point out that the domains are merged to represent the metamaterial driven by the incident radiation as a simple air-dielectric interface with additional currents. The choice of which domains to merge is entirely discretionary, however choosing to merge domains 1 and 2 leads to equations (Eqs. 15 to 19) that share common features with the well-known Fresnel equations. This provides a convenient method to validate the general approach without going for the full-field comparisons (as in Fig. 4a of the main manuscript).

SUPPLEMENTARY NOTE 2: MULTIPOLE CONTRIBUTION FOR E-FIELD POLARIZED PARALLEL TO THE SLIT

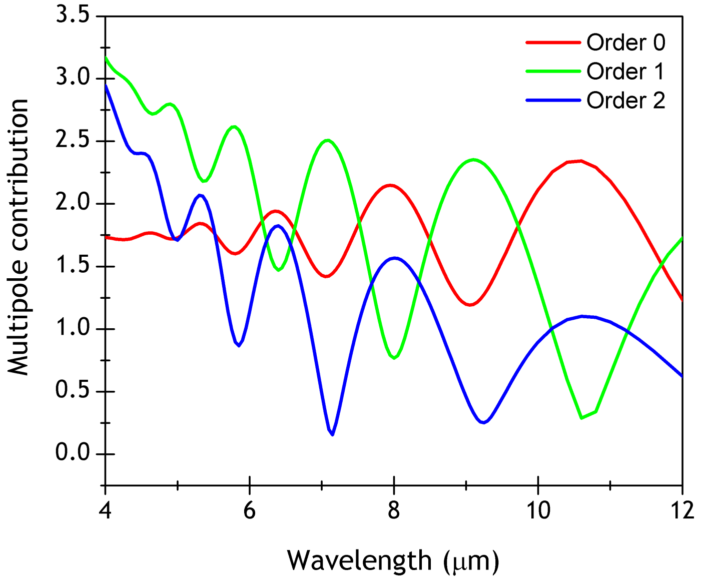

Supplementary Figure 2. The contribution of various multipoles to the (displacement) current excitation induced in the metamaterial by the incident wave polarized parallel to the slits.

SUPPLEMENTARY NOTE 3: PHASE AND AMPLITUDE OF MULTIPOLE CONTRIBUTIONS TO REFLECTION

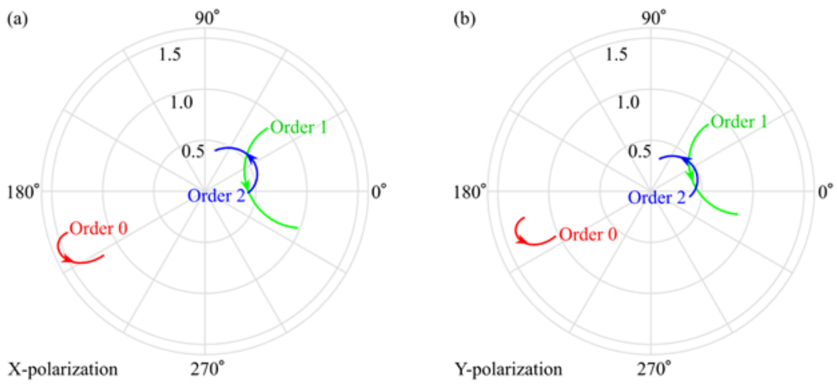

Supplementary Figure 3. Magnitudes and phases of the (dominant) contributions of different multipole orders to the reflection of the metamaterial for the case of x-polarized incident light (a), and y-polarized incident light (b). Each curve corresponds to the range of wavelengths λ_0=7.5µm→8.5µm, with arrows indicating the direction of increasing wavelength. Here z_s=-1200nm.

Figure 3 shows the magnitudes and phases of the contributions the different multipole orders make to the reflection of the metamaterial for the case of excitation along the slit (Fig. S4a), and perpendicular to the slit (Fig. S4b). Note that these quantities are not simply the decomposition of the current excitations in the metamaterial (the M-coefficients described in Sec. S1 and plotted in Fig. 4f of the main paper), instead these are the direct contributions to the reflectivity (i.e), for order l, the plotted quantity is:

$$\frac{-i}{2n_1} \cdot \frac{1}{l!} \left(exp(-i2k_0 n_1 z_s) \cdot \left(\frac{n_1 - n_s}{n_1 + n_s} \right) \cdot (in_1)^l + (-in_1)^l \right) \cdot \frac{M^{(l)}}{\Delta^2}$$

where all the parameters are the same as in Sec. S1. From Fig. S4, one can see that the metamaterial resonance arises as a result of delicate interplay of amplitude and phase variations of three multipole orders with comparable magnitude.

SUPPLEMENTARY NOTE 4: REFLECTION SPECTRA FOR BOTH POLARIZATIONS

For the sake of clarity, we show the experimental reflection spectra of the longest metamaterial nanoslit array (L=4.3 μm) for both polarizations (electric field polarized parallel and perpendicular to the slit length). The differential spectra shown in Figure 3 of the manuscript was determined from these two spectra.

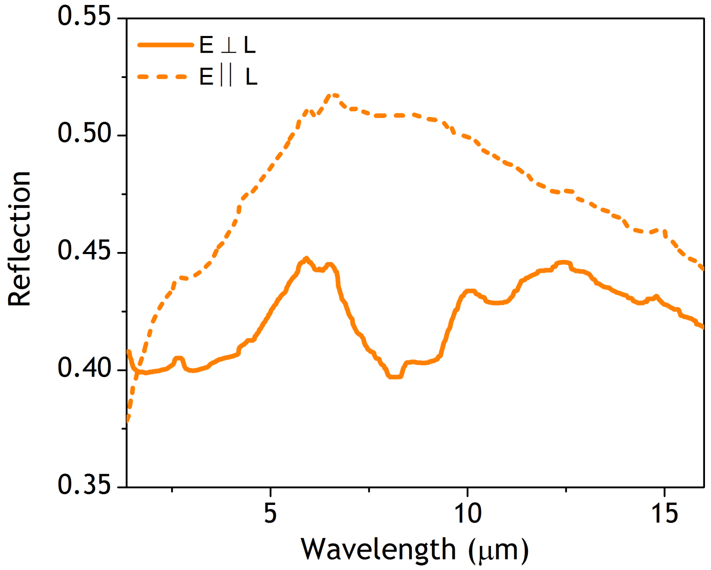

Supplementary Figure 4. Reflection spectra of the longest nanoslit array for incident electric field polarized parallel (dashed line) and perpendicular (solid line) to the slit length.

SUPPLEMENTARY NOTE 5: EVOLUTION OF ELECTRIC AND MAGNETIC FIELDS IN THE METAMATERIAL

To depict the behaviour of the electromagnetic fields within the metamaterial even more clearly, we also show as gifs, the evolution of electric and magnetic fields over half a cycle (0 to π) of the phase, for incident electric field vector polarized perpendicular to the long axis of the slit. The following are the gif files:

- Supplementary Movie 1 - Efield_8p55um.gif (Electric field map and field lines at 8.55 μm).
- Supplementary Movie 2 - Efield_4p35um.gif (Electric field map and field lines at 4.35 μm).
- Supplementary Movie 3 - Hfield_8p55um.gif (Magnetic field map and field lines at 8.55μm).
- Supplementary Movie 4 - Hfield_4p35um.gif (Magnetic field map and field lines at 4.35 μm).

SUPPLEMENTARY REFERENCES

[1] V. Savinov, V. A. Fedotov, and N. I. Zheludev, "Toroidal dipolar excitation and macroscopic electromagnetic properties of metamaterials," *Phys. Rev. B*, vol. 89, p. 205112, 2014.

[2] Assuming time-harmonic fields with time-evolution given by $f(t) = f(0)\exp(+i\omega t)$.

[3] We have replaced $\nabla^2 \to \partial_z^2$ since the solution is expected to be invariant under 'inplane' shifts of the metamaterial.

[4] Note that ρ is *not* the reflection amplitude, since the Υ-integral also contributes to back-propagating wave. Instead, ρ is the additional degree of freedom that will be needed to stitch up the domains 1&2 and 3.

[5] Simple estimates show that $\mathcal{M}^{(l)}/\Delta^2$ should be order of unity or smaller for all l (possibly complex-valued).

[6] V. M. Dubovik and A. A. Cheshkov, "Multipole expansion in classical and quantum field theory and radiation," *Sov. J. Part. Nucl.*, vol. 5, p. 318, 1975.

[7] V. M. Dubovik and V. V. Tugushev, "Toroid moments in electrodynamics and solid-state physics," *Phys. Rep.*, vol. 187, p. 145, 1990.

[8] E. E. Radescu and G. Vaman, "Exact calculation of the angular momentum loss, recoil force, and radiation intensity for an arbitrary source in terms of electric, magnetic, and toroid multipoles," *Phys. Rev. E*, vol. 65, p. 046609, 2002.
