# Supplementary figures and images for: Infrared dielectric metamaterials from high refractive index chalcogenides
Source: Nat Commun. 2020 Apr 3;11:1692. doi: 10.1038/s41467-020-15444-0 (PMC7125163; doi:10.1038/s41467-020-15444-0)

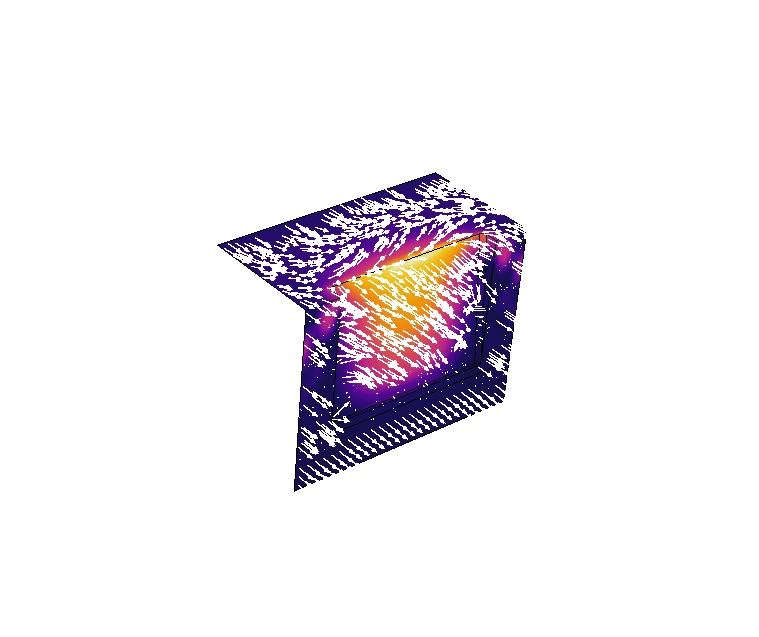

Supplement: Supplementary file 2 — Supplementary Movie 1 [file 41467_2020_15444_MOESM2_ESM.gif]

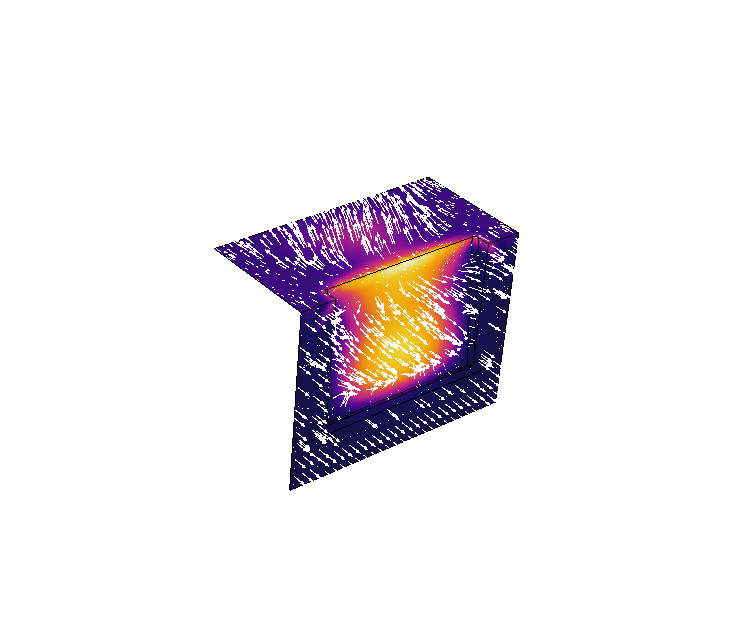

Supplement: Supplementary file 3 — Supplementary Movie 2 [file 41467_2020_15444_MOESM3_ESM.gif]

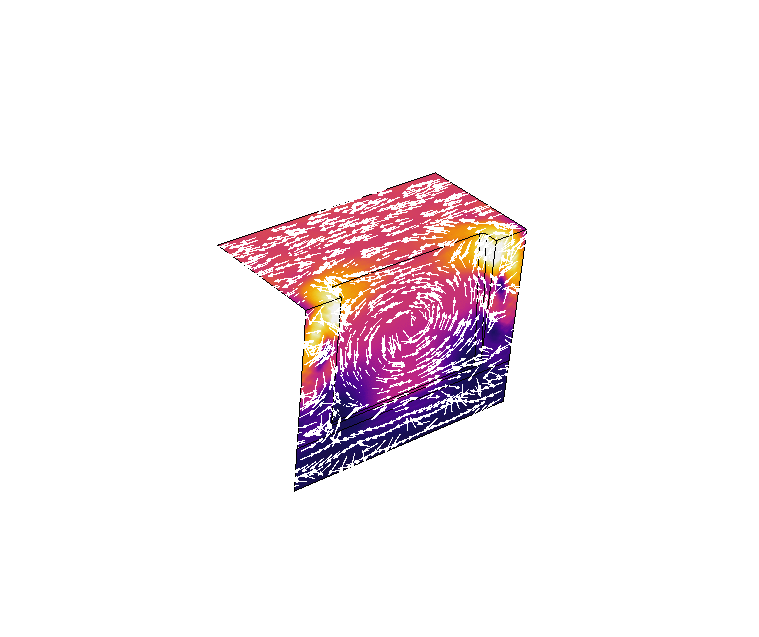

Supplement: Supplementary file 4 — Supplementary Movie 3 [file 41467_2020_15444_MOESM4_ESM.gif]

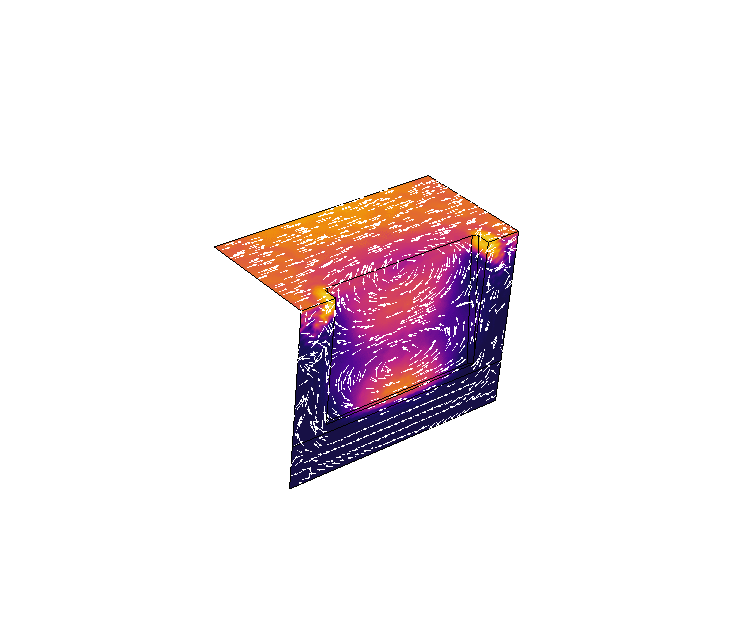

Supplement: Supplementary file 5 — Supplementary Movie 4 [file 41467_2020_15444_MOESM5_ESM.gif]
