# Supplementary material for: Infrared dielectric metamaterials from high refractive index chalcogenides
Source: Nat Commun. 2020 Apr 3;11:1692. doi: 10.1038/s41467-020-15444-0 (PMC7125163; doi:10.1038/s41467-020-15444-0)
Supplement: Supplementary file 6 — Description of Additional Supplementary Files [file 41467_2020_15444_MOESM6_ESM.pdf]

Title: Supplementary Movie 1
Description: Efield_8p55um.gif Description: Evolution of electric field within the metamaterial at the fundamental resonance wavelength of 8.55 μm over half a cycle (0 to π) of the phase. Incident light is polarized perpendicular to the length of the slit. The resulting electric field configuration resembles that of an electric dipole oriented across the slit. 2.

Title: Supplementary Movie 2
Description: Efield_4p35um.gif Description: Evolution of electric field within the metamaterial at the second-order resonance wavelength of 4.35 μm over half a cycle (0 to π) of the phase. Incident light is polarized perpendicular to the length of the slit. The resulting electric field configuration resembles that of two electric dipoles oriented across the slit in opposite directions indicative of a second order resonance. 3.

Title: Supplementary Movie 3
Description: Hfield_8p55um.gif Description: Evolution of magnetic field within the metamaterial at the fundamental resonance wavelength of 8.55 μm over half a cycle (0 to π) of the phase. Incident light is polarized perpendicular to the length of the slit. The resulting magnetic field configuration is characterized by a circulating behavior with a single vortex. 4.

Title: Supplementary Movie 4
Description: Hfield_4p35um.gif Description: Evolution of magnetic field within the metamaterial at the second-order resonance wavelength of 4.35 μm over half a cycle (0 to π) of the phase. Incident light is polarized perpendicular to the length of the slit. The resulting magnetic field configuration is characterized by a circulating behavior with two vortices indicative of a second-order resonance.
